# Role of Epithelial-to-Mesenchymal Transition of Retinal Pigment Epithelial Cells in Glaucoma Cupping

**DOI:** 10.3390/jcm12072737

**Published:** 2023-04-06

**Authors:** Eabha O’Driscoll, Emily Hughes, Mustapha Irnaten, Markus Kuehn, Deborah Wallace, Colm O’Brien

**Affiliations:** 1 Department of Ophthalmology, Mater Misericordiae University Hospital, D07 R2WY Dublin, Irelandmustapha.irnaten2@ucd.ie (M.I.); 2 Department of Ophthalmology and Visual Sciences, University of Iowa Carver College of Medicine, University of Iowa, Iowa City, IA 52240, USA; 3 School of Medicine, University College Dublin, D04 V1W8 Dublin, Ireland

**Keywords:** glaucoma, peripapillary atrophy, epithelial mesenchymal transition, retinal pigment epithelium cell

## Abstract

Optic nerve head (ONH) cupping is a clinical feature of glaucoma associated with extracellular matrix (ECM) remodelling and lamina cribrosa (LC) fibrosis. Peripapillary atrophy (PPA) occurs commonly in glaucoma, and is characterised by the loss of retinal pigment epithelium (RPE) adjacent to the ONH. Under pro-fibrotic conditions, epithelial cells throughout the body can differentiate into fibroblast-like cells through epithelial-to-mesenchymal transition (EMT) and contribute to ECM fibrosis. This is investigated here in the context of glaucoma and PPA. Human-donor ONH sections were assessed for the presence of the RPE cell-specific marker RPE65 using immunofluorescence. We examined the EMT response of ARPE-19 cells to the following glaucoma-related stimuli: cyclic mechanical stretch, mechanical stiffness, transforming growth factor beta (TGFβ), and tumour necrosis factor alpha (TNFα). The gene expression was measured using the PCR of the epithelial tight junction marker zona occludens 1 (ZO-1) and the mesenchymal markers alpha smooth muscle actin (αSMA) and vimentin. A scratch assay was used to assess the ARPE-19 migration. Significant RPE-65 staining was demonstrated in the glaucomatous ONH. The cyclic stretching and substrate stiffness of the ARPE-19 cells caused a significant decrease in ZO-1 (*p* = 0.04), and an increase in αSMA (*p* = 0.04). The scratch assays demonstrated increased migration of ARPE19 in the presence of TNFα (*p* = 0.02). Furthermore, ARPE-19 cells undergo an EMT-like transition (gain of αSMA, loss of ZO-1 and increased migration) in response to glaucomatous stimuli. This suggests that during PPA, RPE cells have the potential to migrate into the ONH and differentiate into fibroblast-like cells, contributing to glaucomatous ONH cupping.

## 1. Introduction

Glaucoma encompasses a group of optic neuropathies and is one the leading cause of irreversible visual impairment and blindness worldwide. It is characterised by optic nerve head (ONH) cupping and visual-field defects. In addition to ONH cupping, its features include the loss of the retinal ganglion cells and the thinning of the retinal-nerve-fiber layer (RNFL). Frequently in glaucoma, peripapillary atrophy (PPA) is observed. In this condition, there is a loss of retinal pigment epithelium (RPE) cells in a crescentic distribution around the ONH [1,2]. While this is also seen in some normal eyes, as well as in myopia, it is found more commonly and is larger in size in eyes with glaucomatous optic neuropathy [3,4]. Beta-zone atrophy (the sub-type most commonly associated with glaucoma) is characterised by bare sclera with exposed choroidal vessels, and Bruch’s membrane is devoid of RPE [1,5]. The progression, conformation, and temporal changes in PPA correlate with the rate and nature of progression of visual-field deterioration in glaucoma [2]. However, little is yet known about the aetiology of PPA or the fate of RPE cells during the process of PPA.

As mentioned above, ONH cupping is a core characteristic of glaucoma. It occurs as changes in the lamina cribrosa (LC), the sieve-like meshwork of connective tissue that supports the optic-nerve axons as they leave the eye. The LC becomes compressed and bowed backwards [6]. This dynamic process of fibrotic change in the LC can be partly explained by a remodeling of the extracellular matrix (ECM) [7]. Optic-nerve-head astrocytes and LC cells have been implicated as mediators of the maladaptive changes in the collagen profile of the ONH [8,9].

Increased levels of pro-fibrotic cytokines, notably transforming growth factor β (TGFβ) and tumor necrosis factor α (TNFα), have been implicated in ONH remodeling in glaucoma [10,11]. Work from our laboratory shows an upregulation of TGFβ global gene-expression analysis in glaucomatous human LC-cell explant cultures compared with normal samples, as well as an upregulation in genes with fibrogenic potential (e.g., collagen I) when normal LC cells are treated with TGFβ [9,12]. Mechanical cyclical stretching has been used successfully as an in vitro stimulus in LC cells and astrocytes, inducing ECM-gene-expression changes associated with the pathophysiology of cupping [13,14].

The process of epithelial-to-mesenchymal transition (EMT) has been implicated in a number of fibrotic disease states, including pulmonary fibrosis [15], renal fibrosis [16], and specifically in ophthalmology, the development of cataracts and proliferative vitreoretinopathy (PVR) [17,18,19]. The EMT involves a decreased expression of epithelial cell markers, such as Zona-Occludens 1 (ZO-1), the increased expression of the mesenchymal markers alpha smooth muscle actin (αSMA) and vimentin, and the increased ability of cells to migrate [16,20]. The EMT can be induced by exposure to the pro-fibrotic growth factors TGFβ [16] and TNFα [21] and by mechanical stress [15]. Furthermore, αSMA has been found to be upregulated in numerous in vitro studies when fibroblasts are treated with TGFβ and, subsequently, to adopt the myofibroblast migratory phenotype [22,23,24]. In addition, during the stimulation of epithelial cells grown on stiff substrates, simulating mechanical stress, EMT took place [16,20]. By no means is EMT a novel aspect of fibrosis. Importantly, with regards to RPE, this has been shown to occur in proliferative vitreoretinopathy (PVR) [18,19]. Retinal pigment epithelium cells were present in the contractile membranes of PVR, with a fibroblast-like phenotype, according to which the RPEs’ intermediate filament profiles were altered [18]. The process of EMT has never been explored in the context of ONH cupping and the LC.

The purpose of this investigation was to determine the potential involvement of RPE cells in LC fibrotic changes via the mechanism of EMT. The first objective of our study was to determine whether there was immunofluorescence-based evidence of RPE-cell expression in the glaucoma ONH. In parallel, we studied the in vitro responsiveness of RPE cells (ARPE19) in terms of EMT-like transition potential following exposure to cyclical stretching, biomechanical stiffness, and the cytokines TGFβ and TNFα. We hypothesise that RPE cells contribute to LC fibrosis by undergoing EMT and migrating to the ONH in glaucoma.

## 2. Materials and Methods

### 2.1. Cell Culture

The ARPE-19 is an immortalised, non-transfected human cell line that spontaneously arose from cultures of human retinal pigment epithelium [25,26] and were obtained from the American Type Culture Collection (CRL-2302). The ARPE-19 cells were cultured in Dulbecco’s Modified Eagle’s Medium (DMEM)/Ham’s F12 1:1 (Sigma D6421) supplemented with 10% (*v*/*v*) fetal bovine serum (Sigma, Wicklow, Ireland), 50 U/mL penicillin, 50 μg/mL streptomycin, and 2 mmol L-glutamine in plastic culture flasks. Cells were used between passages 21 and 30. All experiments were carried out in triplicate.

### 2.2. Cell Culture on Stiffened Substrates

The RPE cells were seeded at a low density of 2000 cells/cm^2^ on commercially available collagen-1 coated polyacrylamide hydrogel substrates (Softwell, Matrigen Products, Cell Guidance Systems, Cambridge, UK) with substrate stiffness levels of 4 kPa (approximating physiological LC stiffness) and 100 kPa (approximating glaucomatous LC stiffness). The differential expression of ZO-1, αSMA, and vimentin in RPE cells grown under these stiffness conditions was examined by qRT-PCR.

### 2.3. Cyclical Cell Stretching

The Flexercell Tension Plus FX-4000T (Dunn Labortechnik, Germany) was used as in a previous study to apply cyclical mechanical stretching to normal ARPE-19 cells [13]. Vacuum pressure was applied to 6-well flexible-bottom laminin matrix-bonded-growth-surface BioFlex™ culture plates (8.25 × 12.5 cm) (BF-3001L Dunn Labortechnik, Germany). The ARPE-19 cells were maintained as described above. When cells reached confluency, medium was replaced with serum-free DMEM/Ham’s F12 for 24 h. Cells were subjected to mechanical stretching followed by relaxation at 60 cycles/min (60 Hz) for 48 h with 15% elongation [13]. Control cells were prepared in the same way on an adjacent plate, but not exposed to stretching. Following stretching, the ARPE-19 cells were released from the laminin plates using trypsin EDTA.

### 2.4. RPE65 Immunofluorescence

The posterior poles of formalin-fixed human-donor eyes were obtained from the Lions Eye Research Institute for Transplant and Research (Tampa, FL, USA). The ocular tissue was embedded in paraffin wax and sectioned at 5 μm through the LC region of the ONH. For RPE65 immunofluorescence, slides were cleared in xylene and rehydrated, and antigen retrieval was performed by boiling for 10 min in a 0.01-M citrate buffer (pH 6.0). A 1:10 solution of goat serum in PBS was applied for 1 h to block non-specific binding. Slides were incubated overnight at 4 °C with 1:250 dilution of mouse monoclonal anti-RPE65 antibody (Abcam, 401.8B11.3D9), followed by incubation in the dark for 1 h with 1:1000 dilution goat anti-mouse IgG-Fab specific FITC conjugate (F2653 Sigma Aldrich Ireland). Images were captured using an Olympus BX51 fluorescent microscope and associated CellSens software (Olympus). Images were assessed qualitatively for any comparative differences in RPE65 expression between the LC of non-glaucomatous and glaucomatous ONHs.

### 2.5. RNA Extraction, cDNA Synthesis, and Quantitative Polymerase Chain Reaction (PCR)

Primers were identified using Primer-BLAST (www.ncbi.nlm.nih.gov, access date: 24 February 2018) and created using a PCR primer-design tool (Eurofins Genomics). Primers were required to have a guanine–cytosine (GC) content of 40–60% to improve stability and were selected to span an exon–exon junction to prevent amplification of genomic DNA. They were expected to have a melting temperature of 52–63 °C. Lyophilised primers were reconstituted in UltraPure distilled H_2_O (Sigma, Dublin, Ireland) at a final stock-solution concentration of 100 μM and stored at −20 °C. Primer sequences for genes used are shown in Table 1.

Total RNA was extracted from cultured human RPM cells using TRIzol reagent (Molecular Research Centre, Dublin, Ireland) according to standard protocols, and RNA was quantified using a NanoDrop ND-1000 spectrophotometer (Labtech, Uckfield, UK). Concentration of RNA was given as μg/μL. The absorbance values for 260/280 and 260/230 ratios were also included in analysis to assess the DNA, RNA, and nucleic-acid purity of the samples. The cDNA was synthesised from up to 2 μg total RNA reverse transcription according to the manufacturer’s instructions. Briefly, a mixture of 1 µL oligo dT (Sigma, Ireland), 1 µL deoxynucleotides (dNTPs) (Sigma, Ireland), and 2 μL of 10× AMV reverse-transcriptase buffer (Sigma, Wicklow, Ireland) and 4μL RNA was heated to 70 °C for 10 min and transferred to ice for 2 min. In total, 0.5 μL of enhanced avian reverse transcriptase eAMV was added and the tube transferred to the thermocycler. The cycle was 45 °C for 90 min, 90 °C for 2 min, cooling to 4 °C, and storage at −20 °C.

### 2.6. Real-Time RT-PCR

The cDNA was assayed in triplicate using the Rotorgene 3000 Real-Time RT-PCR instrument. Quantitative PCR (qPCR) was performed with a Roche LightCycler 480, using SYBR Green Master I (Roche, Dublin, Ireland), according to the manufacturer’s instructions. Amplification of a single product of correct size was confirmed by agarose-gel electrophoresis and/or melting-curve analysis. The QuantiFast Probe Assay (Qiagen, Germantown MD, USA) PCR probes were as follows: ZO-1 (tight junction protein-1)—QF00314552, αSMA—QF00121849, 18S—QF00530467. These were used according to manufacturer’s instructions. Changes in gene expression were quantified relative to the control cDNA. Expression of 18S was used to normalise threshold cycle values (cT) in each PCR cycle. Changes in gene expression were calculated using Delta cT equation—2^−ΔcT^ [27].

### 2.7. Cell-Viability/Crystal-Violet Assay

Cells were fixed in 96% ethanol at room temperature for 10 min, and then stained with 0.05% crystal violet in 20% ethanol for 30 min. Surplus dye was removed, and cell-associated dye was dissolved in 0.1% acetic acid solution (in 50% ethanol). Absorbance at 585 nM was measured. Cell viability was expressed as a percentage relative to control.

### 2.8. Cell Migration

Cells were grown to confluency, after which 55,000 cells/well were transferred to 2-well glass-chamber slides (Lab-Tek 2-chamber slides, C6557). Cells became 90% confluent after 24 h, and medium was replaced with serum-free medium. After 24 h, the cell monolayer was scratched using a p200 pipette tip. Either TGFβ1 (10 ng/mL) or TNFα (10 ng/mL) [28] was added to one well, while the other well was used as a control [10,11]. After culturing for a further 24 h, cells were fixed in cold 4% paraformaldehyde for 15 min, rinsed in cold PBS, and blocked for 2 h at room temperature. Cells were mounted using Prolong Gold antifade DAPI (nuclear stain, 4′,6-diamidino-2-phenylindole, Invitrogen P36935) and sealed with a coverslip before imaging. To assess cell migration, each scratch wound was imaged at three points perpendicular to the scratch wound at 10× magnification. Migration was measured by comparing the width of the wound in the growth-factor-exposed cells to a non-growth-factor control. Measurements were made at 5 equidistant points along the length of the scratch image using Photoshop software and averaged.

### 2.9. Cell Proliferation

The ARPE-19 cells were cultured on glass-bottomed two-well-chamber slides, as described for cell migration. After 24 h, scratch wounds were applied to the cell monolayer using a p200 pipette tip, as described above. Either TGFβ1 (10 ng/mL) or TNFα (10 ng/mL) was added to one well, the other well was used as a control. Cells were cultured for a further 24 h, after which they were fixed in cold 4% paraformaldehyde for 15 min. Cells were rinsed in cold PBS, and then blocked with blocking buffer for 2 h at room temperature. Additionally, Ki67 immunofluorescence was used to assess cell proliferation. Cells were incubated with a primary rabbit polyclonal antibody to Ki67 (1:75 dilution, Santa Cruz BT sc15402) in a humidified chamber overnight at 4 °C. Slides were then washed with PBS and a secondary antibody (1:300, Rabbit Texas Red, Invitrogen 2767) was added for one hour at room temperature. Cells were washed and mounted using Prolong Gold antifade DAPI (nuclear stain, 4′,6-diamidino-2-phenylindole, Invitrogen P36935) and cover-slipped before imaging. The number of Ki-67-positive cells was expressed as a percentage of DAPI-positive cells. This was ascertained by counting cells positively stained for Ki-67 and DAPI in a given field (with 15≤ cells, at 20× magnification):(Number of cells Ki-67 positive ÷ Number of cells DAPI positive) × 100. This allowed standardization between scratch experiments. Measurements were taken at three locations along each scratch for each experiment.

### 2.10. Statistical Analysis

Triplicate results were obtained for each cell-culture experiment with three independent experiments and three technical replicates performed for each. A Student’s *t*-test was used to determine statistical significance between two samples (normal versus experimental stimulus). Statistical significance was set at *p* ≤ 0.05. Results were graphed on a bar chart as mean ± SD (standard deviation).

## 3. Results

### 3.1. RPE65 Is Markedly Expressed throughout the Lamina-Cribrosa Region in Glaucoma

**Figure 1:**
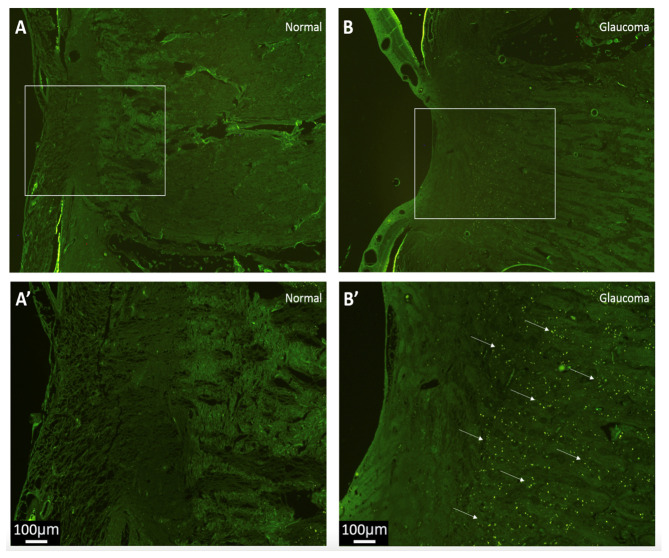
RPE-65 immunofluorescence of longitudinal sections through the laminar region of the human optic nerve from normal (**A**,**A’**) and glaucoma (**B**,**B’**) donors. Due to endogenous autofluorescence, the underlying tissue ultrastructure of the laminar region is visible (green) in both normal (**A**) and glaucoma (**B**) tissue sections, with the white box denoting the magnified LC region photographed in (**A’**,**B’**). A strong positive fluorescent RPE65 signal (bright green dots, indicated with arrows) was seen only in the laminar and post-laminar tissues of the glaucoma donor nerve (**B’**) compared to the same region in the normal donor (**A’**). Scale bar 100 µm.

As illustrated in Figure 1, the expression of the RPE-specific protein marker RPE65 was seen extensively throughout the laminar and post-laminar tissues only in the glaucomatous donor (B’). No positive fluorescent RPE65 signals were observed in the normal LC tissue (A’) or in the negative control slides (primary antibody omission). This difference was qualitative and assessed subjectively.

### 3.2. Cyclic Mechanical Stretching Reduces ZO-1 Expression and Increases αSMA-Gene Expression in ARPE-19 Cells Post-Stretching

**Figure 2:**
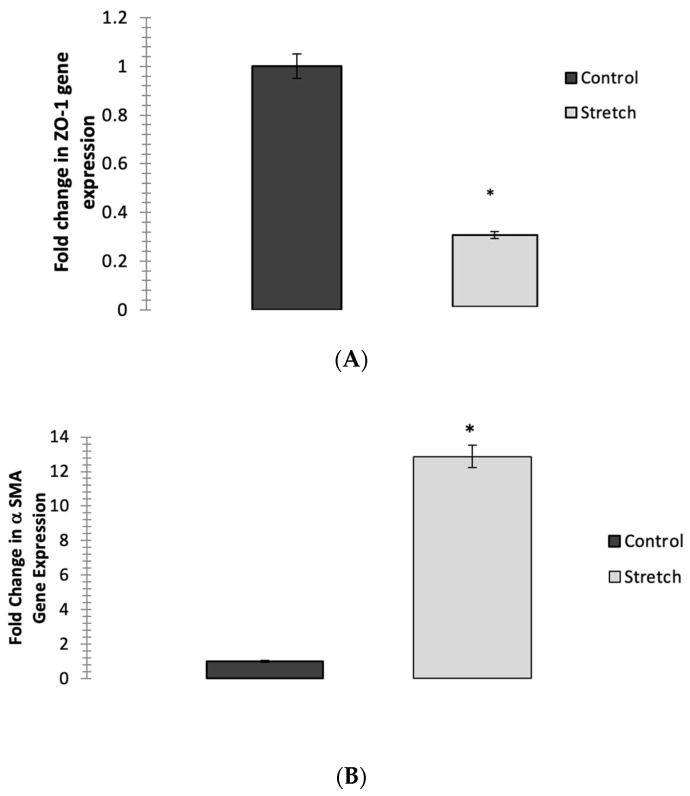
(**A**) Effect of stretching on ZO-1 gene’s EMT-related expression in ARPE-19 cells. Fold change is expressed relative to untreated control after 48 h of cyclical stretching and measured by qPCR. A Student’s *t*-test was used to determine statistical significance between two samples (Control vs. Stretch). The ZO-1 gene expression resulted in fold change of 0.3 ± 0.2 (*p* = 0.04) compared to unstretched control. (**B**) Effect of stretching on αSMA-gene fibrosis-related expression in ARPE-19 cells. Fold change is expressed relative to untreated control after 48 h of cyclical stretching and measured by qPCR. A Student’s *t*-test was used to determine statistical significance between two samples (Control vs. Stretch). The αSMA-gene expression resulted in fold change of 12.6 ± 0.9 (*p* = 0.04) compared to unstretched control. Significantly different values are denoted by asterisks. Data were expressed as mean ± S.D from three biological replicates; * *p* < 0.05.

Two of the hallmarks of EMT are the loss of epithelial (ZO-1) and the gain of mesenchymal markers (αSMA). The expression of the ZO-1 and αSMA genes following the 15% cyclical stretching of ARPE-19 cells for 24 h was measured by qPCR. The cyclical stretching decreased the ZO-1 expression to 0.3 ± 0.2 relative to the control (*p* = 0.04) (Figure 2A). The αSMA expression increased in the stretched cells, to 12.6 ± 0.9 (*p* = 0.04) (Figure 2B).

### 3.3. Increased Biomechanical Stiffness Reduces ZO-1-Gene Expression and Increases Vimentin and αSMA Gene Expression in ARPE-19 Cells

**Figure 3:**
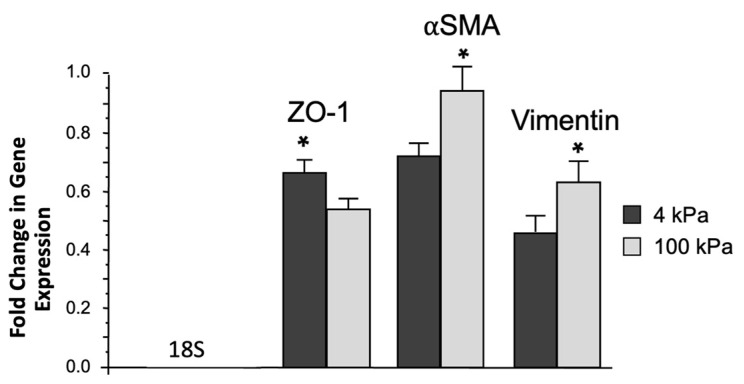
Effect of a stiff substrate on EMT-related gene expression in ARPE-19 cells. Fold change was calculated against the expression of ‘house-keeping’ gene 18S. A Student’s *t*-test was used to determine statistical significance between two samples (soft substrate 4 kPa vs. stiff substrate 100 kPa). The ZO-1-gene expression was significantly decreased when RPE cells were grown on stiff substrate (0.662 ± 0.039 (4 kPa) versus 0.537 ± 0.034 (100 kPa), *p* < 0.05, n = 3), while αSMA-gene expression was upregulated (0.711 ± 0.041 (4 kPa) versus 0.926 ± 0.095 (100 kPa)), and vimentin-gene expression was also enhanced (0.448 ± 0.052 (4 kPa) versus 0.614 ± 0.073 (100 kPa)). Significantly different values are denoted by asterisks. Data were expressed as mean ± S.D from three biological replicates; * *p* < 0.05.

The expression of ZO-1, vimentin, and αSMA following culturing on 4 kPa and 100 kPa collagen-1-coated polyacrylamide hydrogel of ARPE-19 cells was measured by qPCR. Increased stiffness (100 kPa) and decreased ZO-1 expression to 0.3 ± 0.2 compared to soft (4 kPa)-cultured ARPE-19 (*p* = 0.04) were observed (Figure 3). vimentin- and αSMA-gene expression were significantly increased to 12.6 ± 0.9 when the ARPE-19 cells were grown on the stiff substrate (100 kPa) compared to the soft substrate (4 kPa) (*p* = 0.04) (Figure 3).

### 3.4. Effect of TGFβ1 and TNFα on ARPE-19-Cell Migratory Response

**Figure 4:**
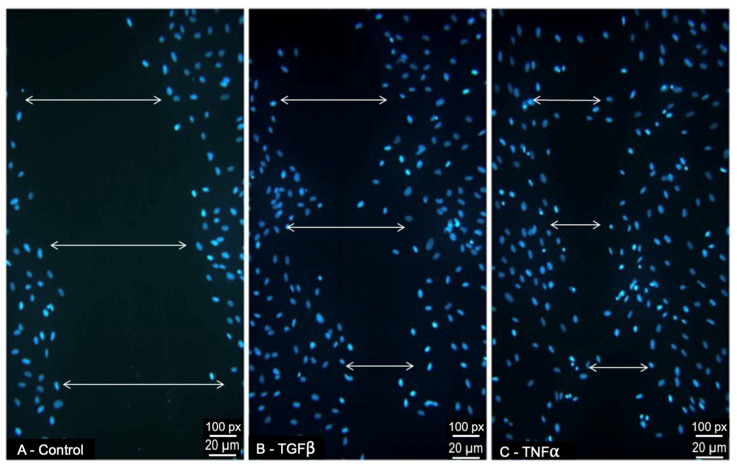
ARPE19-cell migratory response to treatment with pro-fibrotic cytokines (**B**) TGFβ1 (10 ng/mL) and (**C**) TNFα (10 ng/mL) during scratch migration assay. The ARPE19 cells stained with nuclear marker DAPI (blue), 24 h after the infliction of a scratch wound. Migration was calculated as pixels (px)/hour. Scratch wounds, indicated with white arrows, were measured. Control (**A**) scratch wound (serum-free medium only) measured 650 px (±81), (**B**) TGFβ1 scratch wound measured 449 px (±19), and (**C**) TNFα scratch wound measured 362 (±70). (**B**) TGFβ1 (*p* = 0.07) and (**C**) TNFα (*p* = 0.02) induced migration. They caused a similar degree of wound closure to control (n = 3).

The cells that were treated with TGFβ1 (10 ng/mL) or TNFα (10 ng/mL) [28] 24 h post-scratching were assessed for migration (Figure 4). The migration was calculated in pixels/hr. The TGFβ1 caused the migration of the ARPE-19 cells into the scratch wound, with the wound measuring 449 pixels (px) (±19, *p* = 0.07) at 24 h compared to the control wound of 650 pixels (±81) (Figure 4). The TNFα caused a significant increase in migration, with wounds measuring 362 pixels (±70, *p* = 0.02) at 24 h. A narrower wound width compared to the control implies the migration of the cells into the wound.

### 3.5. ARPE-19-Cell Proliferation in Response to Treatment with TGFβ1 and TNFα during Scratch Migration Assay

**Figure 5:**
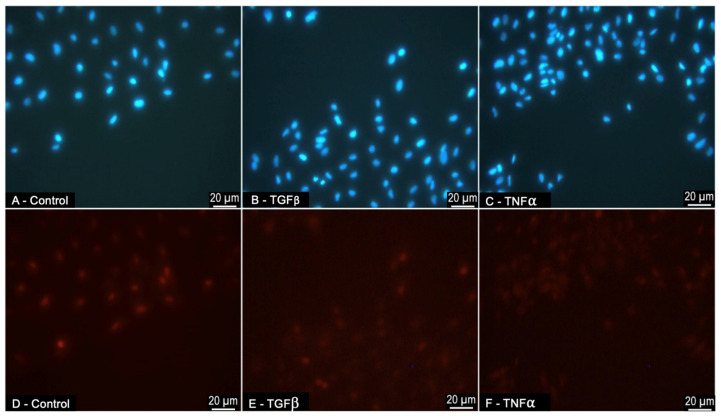
ARPE19-cell proliferation in response to treatment with pro-fibrotic cytokines (**B**,**E**) TGFβ1 and (**C**,**F**) TNFα during scratch migration assay. The ARPE19-cell immunofluorescence at the border of the scratch wound (20× magnification). (**A**–**C**) Cells were stained for nuclear marker DAPI (blue) and (**D**–**F**) cell-proliferation marker Ki-67 (red). The number of Ki-67-positive cells was expressed as a percentage of DAPI-positive cells. This was ascertained by counting cells positively stained for Ki-67 and DAPI in a given field (with 15≤ cells, at 20× magnification):(Number of cells Ki-67 positive ÷ Number of cells DAPI positive) × 100. There were no significant differences between the levels of proliferation between the groups (*p* > 0.05). (n = 3).

The cells that were assessed for migration by scratch assay were stained with proliferation marker Ki-67 and nuclear marker DAPI (Figure 5). The cells at the border of the scratch wound were imaged. There were no significant differences (*p* > 0.05) between the levels of proliferation present in the three scratch experiments, control 32% (±10%), TGFβ 45% (±11%), and TNFα 40% (±7%). There was no statistical significance in the number of cells proliferating in the cytokine-treated cells compared to the controls, demonstrating that any migration was true, and not an artefact of proliferation.

## 4. Discussion

Glaucomatous PPA is clinically significant to the progression of ONH damage and visual-field loss, but whether this is a co-variant association or whether there may be a causative association in either direction is yet to be elucidated. The overarching hypothesis in our study centres on the potential for RPE cells to contribute to ONH remodelling through migration into the ONH and transform into a mesenchymal phenotype as a consequence of injury-associated EMT. The studies we conducted provide observation-based support for this hypothesis by demonstrating increased RPE-65 localised in the glaucomatous ONH and the capacity for cultured RPE cells to undergo EMT in vitro.

Human normal and glaucomatous ONHs were examined for evidence of the RPE-cell-associated visual-cycle protein RPE-65. The RPE-65 was retained in RPE-cell cultures shown to have undergone EMT, making it a reliable marker of this cell type [29]. Alongside the positive staining of the RPE in situ, there was increased positive staining within the glaucomatous ONH, as visualised by immunofluorescence. Anatomically, the junction between the intermediate tissue of Kuhnt and the border tissue of Elschnig may represent a route of migration for the RPE into the ONH [30]. This result was qualitative in nature and presents a novel finding, as there is no published evidence for the expression of RPE65 within the ONH. A further step in our research would be to quantify this difference.

In order for RPE cells to play a part in the fibrotic cupping of the LC in glaucoma, EMT must occur. This involves the adoption of a migratory phenotype, a loss of epithelial markers, and a gain in mesenchymal markers [15,16,22,23,24]. This has been demonstrated in vitro in renal and pulmonary fibrosis using the same stimuli relevant to the ONH in glaucoma, namely TGFβ and cyclical stretch [15,16]. Renal tubular epithelial cells transition to a mesenchymal fibroblast-like phenotype through a number of steps: the loss of cell adhesion, actin reorganisation, basement-membrane disruption, and cell migration [16]. This transition has also been shown to occur in glaucoma, in which EMT takes place in Schelmm’s canal and TM, which are essential anatomical sites for the drainage of aqueous humor and IOP regulation via glaucomatous stimuli [31,32]. Importantly, with regards to RPE, this has been shown to occur in proliferative vitreoretinopathy (PVR) [18,19]. Retinal pigment epithelium cells were present in the contractile membranes of PVR, with a fibroblast-like phenotype, according to which the RPEs’ intermediate filament profiles were altered [18]. Therefore, RPE cells have the potential to contribute to pathological processes via EMT. To date, this has not been demonstrated in the LC of glaucomatous ONH. Our current in vitro study shows that epithelial marker ZO-1 is indeed upregulated in ARPE19 control samples and in ARPE19 soft substrate (4 kPa)-cultured cells prior to any introduction of glaucomatous stimuli. After the introduction of the known glaucomatous stimuli, TNFα, TGFβ, 15% cyclical stretching, and increased stiffness (100 kPa), there is a clear change in the gene signatures of ARPE19 cells, with a significant loss of ZO-1 and the upregulation of the mesenchymal markers vimentin and αSMA. Through the EMT, we showed that the ARPE-19 adopted a migratory phenotype stimulated by TGFβ or TNFα, which caused similar degrees of wound closure on the scratch assay, a result that could not be attributed to increased cell proliferation, as shown in our proliferation assay. This is very interesting, as TGFβ levels are increased in the glaucomatous optic nerve [10], and TNFα levels increased in the retina in glaucoma [11].

In summary, this work demonstrated evidence of RPE-cell protein within the ONH of human glaucoma donors. In parallel, the human RPE cell line (ARPE-19) was shown to undergo an EMT-like transition in response to glaucomatous stimuli. The cells were shown to have the altered phenotype of increased myofibroblastic gene expression, decreased epithelial gene expression, and enhanced migratory ability. With its consideration of the pathophysiology of glaucoma, our in vitro study suggests that RPE-cell EMT and consequent migration to the ONH may certainly contribute to the dynamic process of fibrotic change in the LC and add to the remodeling of the ECM. The application of this knowledge to the loss of RPE cells (i.e., beta zone PPA) in vivo in eyes with glaucoma may shed new light on the process of peripapillary atrophy.

## Figures and Tables

**Table 1:** Primer sequences for quantitative real-time RT-PCR.

Gene Name	Forward	Reverse
18S	5′-GTAACCCGTTGAACCCCATT	5′-CCATCCAATCGGTAGTAGCC
ZO-1	5′-CCTCTTCCTGATGGATGGGAAC	5′-TATTCCGCATTGCCTGCCG
Vimentin	5′-TTCTGTACGCAGGTGATTGG	5′-CATGTTCAGCTTTGTGGACC
αSMA	5′-AAAGCTTCCCAGACTTCCGC	5′-TTCTTGGGCCTTGATGCGAA

## Data Availability

Data is contained within the article.
